# Doege Potter syndrome presenting as multiple fibrous tumours of the chest

**DOI:** 10.1093/icvts/ivac089

**Published:** 2022-04-05

**Authors:** Britton B Donato, Marisa A Sewell, Ayan Sen, Staci E Beamer

**Affiliations:** 1 Department of Surgery, Mayo Clinic Hospital, Phoenix, AZ, USA; 2 Department of Surgery, Oregon Health & Science University, Portland, OR, USA; 3 Department of Critical Care Medicine, Mayo Clinic Hospital, Phoenix, AZ, USA

**Keywords:** Pleural mass, Paraneoplastic hypoglycaemia, Solitary fibrous tumour

## Abstract

Doege Potter syndrome is a rare condition causing non-islet cell paraneoplastic hypoglycaemia associated with fibrous tumours, which can be both benign and malignant. The vast majority are solitary and located within the chest. Non-islet cell tumour-induced hypoglycaemia, as in Doege Potter syndrome, is quite rare and occurs around 4 times less often than islet cell-associated paraneoplastic hypoglycaemia. We present a case of Doege Potter syndrome with severe hypoglycaemia in conjunction with multiple recurrent fibrous tumours of the lung and pleura.

## INTRODUCTION

Doege Potter syndrome is a rare condition in which affected patients suffer from non-islet cell paraneoplastic hypoglycaemia in conjunction with fibrous tumours. Most often, these patients present with a solitary fibrous tumour (SFT), usually in the chest. We present a case of Doege Potter syndrome presenting as severe hypoglycaemia in conjunction with multiple recurrent fibrous tumours of the lung and pleura [1].

## CASE

**Figure 1:**
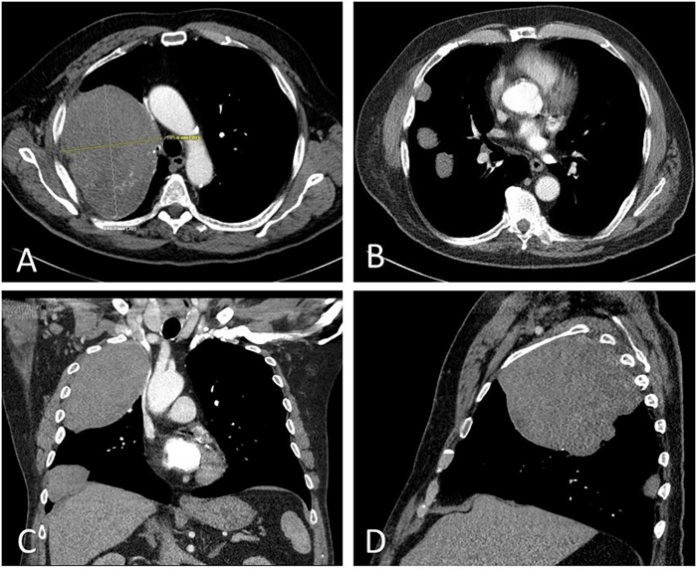
Computed tomography with axial (**A** and **B**), coronal (**C**) and sagittal (**D**) images showing 4 solid right pleural masses, the largest measuring 14 cm × 10 cm × 12 cm with heterogenous enhancement.

**Figure 2:**
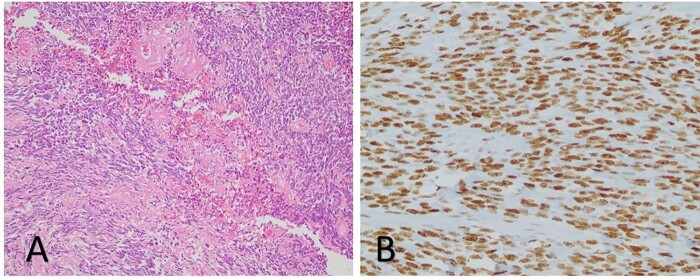
(**A**) (H&E 10×) Typical low-magnification appearance of a fibrous area with variable cellularity, collagenous stroma and rounded hyalinized blood vessels. (**B**) Immunohistochemistry (STAT6, 20×), strong and diffuse nuclear staining in the tumour cells.

This patient is an 81-year-old male with a history of insulin-dependent diabetes mellitus type 2 and recurrent SFTs of the chest who presented to us with 6 months of symptomatic, progressive hypoglycaemia and was ultimately diagnosed with Doege Potter syndrome. In 1989 and 2009, he underwent resection of benign SFTs in the right chest, both of which were via right thoracotomy. Final pathology for both resections demonstrated benign SFT. Following resection, routine postoperative surveillance demonstrated recurrence of suspected fibrous tumours. Due to his age and lack of symptoms, the pleural masses were actively monitored with annual computed tomography (CT) scans. Ten years following his last surgery, he developed increasing episodes of symptomatic hypoglycaemia despite a history of type II diabetes. Because of his progressive hypoglycaemia, he was started on oral prednisone to maintain euglycaemia. Repeat CT scan revealed continued growth of a right apical lung mass and several smaller pleural masses (Fig. 1). Preoperative workup revealed low serum levels of insulin (<1 uIU/l, reference range 2–25) and C-peptide (0.3 ng/ml, reference range 1.1–4.4), suggesting that the hypoglycaemia was associated with an insulin-like peptide not detected on initial screening. The patient’s history of recurrent fibrous tumours of the chest, along with 6 months of severe hypoglycaemia in conjunction with low serum insulin, led to a diagnosis of Doege Potter syndrome. He underwent a redo right thoracotomy with the resection of several pleural masses, right middle and lower lobe wedge resections, right upper lobectomy and mediastinal lymphadenectomy. Final surgical pathology was consistent with SFT (Fig. 2). All lymph nodes were negative for malignancy and an R0 resection was achieved.

Following surgery, the patient’s hypoglycaemia resolved. He was discharged home on postoperative day 8 and is currently doing well. Surveillance imaging has demonstrated a 14-mm nodule on the right hemidiaphragm, which has been unchanged in size over the last year and he remains asymptomatic.

## DISCUSSION AND CONCLUSION

Doege Potter syndrome is defined as non-islet cell paraneoplastic hypoglycaemia associated with fibrous tumours. These tumours are usually solitary; however, our patient presented with multiple recurrent fibrous tumours of the lung and pleura. Non-islet cell tumour-induced hypoglycaemia, as in Doege Potter syndrome, is quite rare and occurs around 4 times less often than islet cell-associated paraneoplastic hypoglycaemia [2, 3]. These tumours secrete large levels of an incompletely processed precursor of insulin-like growth factor II (IGF-II), which upsets physiological glucose balance and ultimately results in episodes of severe hypoglycaemia coinciding with low serum insulin, C-peptide and IGF-1 [4]. Workup demonstrates episodes of low serum glucose, often <50 mg/dl, associated with low serum insulin, C-peptide and IGF-1. Once non-islet cell tumour-induced hypoglycaemia is suspected, CT imaging studies are used to identify the primary tumour, as it has the greatest preoperative diagnostic value in characterizing intrathoracic fibrous tumours [4].

Evidence suggests that neoplastic non-islet cell tumour hypoglycaemia can be cured by complete *en bloc* resection [3]. Following surgery, our patient quickly returned to his previous glycaemic status, requiring insulin and metformin to treat diabetic-related hyperglycaemia. Though usually associated with a single solitary tumour, our patient presented with multiple SFTs in addition to a history of 2 previously resected asymptomatic SFTs. Patients carrying this diagnosis have an excellent prognosis when identified early, often achieving complete disease remission following tumour removal.

**Conflict of interest:** none declared.
